# Decellularized Matrix from Tumorigenic Human Mesenchymal Stem Cells Promotes Neovascularization with Galectin-1 Dependent Endothelial Interaction

**DOI:** 10.1371/journal.pone.0021888

**Published:** 2011-07-11

**Authors:** Jorge S. Burns, Malthe Kristiansen, Lars P. Kristensen, Kenneth H. Larsen, Maria O. Nielsen, Helle Christiansen, Jan Nehlin, Jens S. Andersen, Moustapha Kassem

**Affiliations:** 1 Molecular Endocrinology Laboratory KMEB, Department of Endocrinology and Metabolism, Odense University Hospital, University of Southern Denmark, Odense, Denmark; 2 Laboratory of Cell Biology and Advanced Cancer Therapies, Department of Oncology, Hematology and Respiratory Disease, University Hospital of Modena and Reggio Emilia, Modena, Italy; 3 Department of Biochemistry and Molecular Biology, Center for Experimental BioInformatics, University of Southern Denmark, Odense, Denmark; 4 Department of Clinical Immunology, Institute of Clinical Research, Odense, Denmark; 5 Stem Cell Unit, Department of Anatomy, College of Medicine, King Saud University, Riyadh, Kingdom of Saudi Arabia; Faculdade de Medicina, Universidade de São Paulo, Brazil

## Abstract

**Background:**

Acquisition of a blood supply is fundamental for extensive tumor growth. We recently described vascular heterogeneity in tumours derived from cell clones of a human mesenchymal stem cell (hMSC) strain (hMSC-TERT20) immortalized by retroviral vector mediated human telomerase (hTERT) gene expression. Histological analysis showed that cells of the most vascularized tumorigenic clone, -BD11 had a pericyte-like alpha smooth muscle actin (ASMA+) and CD146+ positive phenotype. Upon serum withdrawal in culture, -BD11 cells formed cord-like structures mimicking capillary morphogenesis. In contrast, cells of the poorly tumorigenic clone, -BC8 did not stain for ASMA, tumours were less vascularized and serum withdrawal in culture led to cell death. By exploring the heterogeneity in hMSC-TERT20 clones we aimed to understand molecular mechanisms by which mesenchymal stem cells may promote neovascularization.

**Methodology/Principal Findings:**

Quantitative qRT-PCR analysis revealed similar mRNA levels for genes encoding the angiogenic cytokines VEGF and Angiopoietin-1 in both clones. However, clone-BD11 produced a denser extracellular matrix that supported stable *ex vivo* capillary morphogenesis of human endothelial cells and promoted *in vivo* neovascularization. Proteomic characterization of the -BD11 decellularized matrix identified 50 extracellular angiogenic proteins, including galectin-1. siRNA knock down of galectin-1 expression abrogated the *ex vivo* interaction between decellularized -BD11 matrix and endothelial cells. More stable shRNA knock down of galectin-1 expression did not prevent -BD11 tumorigenesis, but greatly reduced endothelial migration into -BD11 cell xenografts.

**Conclusions:**

Decellularized hMSC matrix had significant angiogenic potential with at least 50 angiogenic cell surface and extracellular proteins, implicated in attracting endothelial cells, their adhesion and activation to form tubular structures. hMSC -BD11 surface galectin-1 expression was required to bring about matrix-endothelial interactions and for xenografted hMSC -BD11 cells to optimally recruit host vasculature.

## Introduction

Bone marrow derived hMSC may have a supportive role in tumorigenesis [1], even possibly an ontogenic role in Ewing's sarcomas [2] where angiogenesis and vasculogenesis are prominent. To improve upon existing outcomes (long term survival typically <50%), alternative therapeutic strategies include disruption of how these sarcomas obtain and maintain a blood supply [3]. Since tumorigenic cells can acquire a blood supply via distinct processes, detailed understanding of the specific molecular mechanisms involved is required for appropriate therapeutic strategies. Angiogenesis (new blood vessels from pre-existing vessels), or tumour vasculogenesis (recruitment of bone marrow endothelial progenitor cells to form *de novo* vessels) are influenced by vascular endothelial growth factor (VEGF) [4]. In contrast, VEGF apparently contributed little to a process termed vasculogenic mimicry, when Ewing sarcoma cells themselves contributed to the vascular network [5].

In addition to cellular secretion of angiogenic factors such as VEGF, the production of extracellular matrix contributes to vascularization by a wide range of dynamic mechanisms. Cell signalling is mediated via adhesion receptors such as integrins, sequestered growth factors [6] and mechanical characteristics of the matrix, which combine to influence endothelial cell differentiation, survival, polarity and migration [7]. Moreover, different forms of angiogenesis probably involve different forms of extracellular matrix (ECM) and endothelial-ECM interactions and there is a need for a better understanding of the potential players and their roles [8].

Bone marrow derived hMSC can function as perivascular cells, stabilizing engineered vessels when combined with endothelial cells [9]. Indeed, a consistent perivascular location in a broad range of tissues, has led to the hypothesis that hMSC may have a perivascular origin [10], defining an intimate association with vasculature. We recently described clone-specific heterogeneity in the vascularization of tumours derived from hMSC-TERT20 cells [11],[12]. This tumorigenic model [13] evolved spontaneously from long-term passage of telomerized hMSC [14] that had hitherto retained the phenotype of primary mesenchymal stem cells including multipotent differentiation potential [15]. Thus hMSC-TERT20 clones provided a versatile model for tumour vascularization within the context of a perivascular cell type. Molecular mechanisms governing how the most angiogenic clone recruits vasculature may be broadly relevant for both anti-angiogenic tumor therapy and current investigations regarding the application of mesenchymal stem cells for clinical treatment of ischemia [16]. Here, we show that upon serum starvation, the most angiogenic tumor clone -BD11 produced an extracellular matrix that supported autonomous cord-like cellular reorganisation, resembling the capillary morphogenesis of endothelial cells cultured on Matrigel™. Decellularized -BD11 cell matrix could guide cord-like cellular organisation of seeded endothelial cells and moreover, sufficed to promote neovascularization in an *in vivo* Matrigel™ encapsulated sponge assay. Preliminary characterisation via mass spectrometry of metabolically labelled decellularized matrix identified 50 cell surface proteins known to have a role in angiogenesis. Among them, galectin-1 was expressed in serum-deprived cultures of -BD11 cells and played a key role in decellularized hMSC matrix-endothelial interactions and neovascularization *in vivo*.

## Results

### Potently tumorigenic -BD11 cells survived serum starvation with spontaneous cord morphogenesis

**Figure 1:**
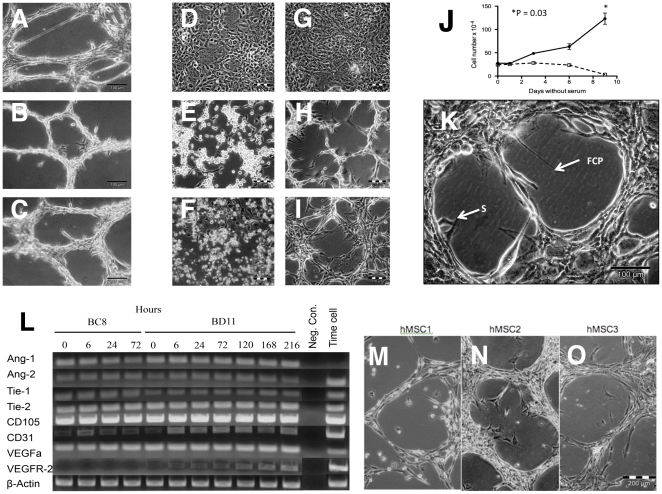
Cord formation of primary hMSC and hMSC-TERT clones. Cord morphogenesis with cell sprouting in *A*: Primary hMSC, *B*: -BC8 and *C*: -BD11 induced by culture on Matrigel® overnight. *D*,*E*,*F*: Phase contrast photomicrographs of -BC8 and *G*,*H*,*I*: -BD11 cells during serum starvation. *D*,*G*: confluent cells before serum starvation for *E*,*H*: 2 days and *F*,*I*: 7 days. *J*, Growth curves for -BC8 (□) and -BD11 (•) in serum free medium, *p<0.05. *K*: -BD11 cells after 72 hours in serum free medium, showing early cell sprouts (S) and a lamellipodium terminating in a focal contact point (FCP) within the lacuna. *L*: RT-PCR analysis of cDNA obtained from serum-starved hMSC-TERT20 clones (time without serum indicated in hours). *M*,*N*,*O*: Independent examples of plastic adherent primary hMSCs spontaneously forming cords when depleted of growth factors for 2 weeks.

The previously described clonal cell lines: hMSC-TERT20-BC8 (-BC8) and -hMSC-TERT20-BD11 (-BD11), showed different tumorigenic potential [11] correlated to the extent of tumour-related vasculature [12]. As seen for primary bone marrow derived hMSC (Figure 1*A*), their vasculogenic phenotype included cord formation on Matrigel (Figure 1*B*, 1*C*). Removing serum from confluent -BC8 (Figure 1*D*) or -BD11 (Figure 1*G*) “cobblestone” monolayers, promptly led to cell proliferation and migration, both clones yielding circular lacunae approximately 40 µm in diameter within 6 hours. Yet within 72 hours, growth of -BC8 cells stopped (Figure 1*J*) and these cells died (Figure 1*F*) whereas -BD11 cells proliferated forming cell cords (Figure 1*I*). Time-lapse photography of -BD11 cells revealed three main phases to branching cell cord network formation (Supplementary Movie S1). Within 24 hours of serum starvation there was extensive mitotic activity. During the 24–48 hour period, cells condensed towards each other, retracting the borders of the growing lacunae. Subsequently, in a consolidation phase, the cords of cells maintained their honeycomb-like network pattern distribution with less cell migration and cell division. The -BD11 viability persisted in replenished serum-free medium for at least three weeks. Occasionally, a sprouting lamellipodium ending with a focal contact point (FCP), projected into the acellular space. Whilst the lacunae circumference increased, the non-dividing cell body retracted towards neighbouring cells, extending the length of the lamellipodium in the process (Fig. 1*K*). Subsequently, a cell could migrate into the lacuna towards its lamellopodial focal contact point and continue its migration. Cell cord morphogenesis was reversible. Upon addition of serum-containing medium the cells re-established a confluent monolayer.

Both serum-starved clones expressed Ang-1, Ang-2, Tie-1, Tie-2, CD31, CD105 and VEGFa mRNA, whereas -BD11 cells also expressed low levels of VEGFR-2 mRNA, increasingly detected during the serum starvation time course (Figure 1*L*). Reflecting low expression, VEGFR-2 or CD31 protein was not readily detected by FACS analysis of -BC8 or -BD11 cells cultured in ECBM-MV2 medium (data not shown). Notably, control TIME endothelial cells did not express Ang-1 but had greater PCR band intensity for Ang-2, Tie-1, CD31 and VEGFR-2.

The cord morphogenesis observed in serum depleted -BD11 and -BC8 cells was sometimes observed in subregions of primary hMSC cultures (Figure 1*M,N,O*), thus this starvation phenotype was expressed by a subset of primary cells.

### -BD11 cells were more angiogenic than clone -BC8 cells

**Figure 2:**
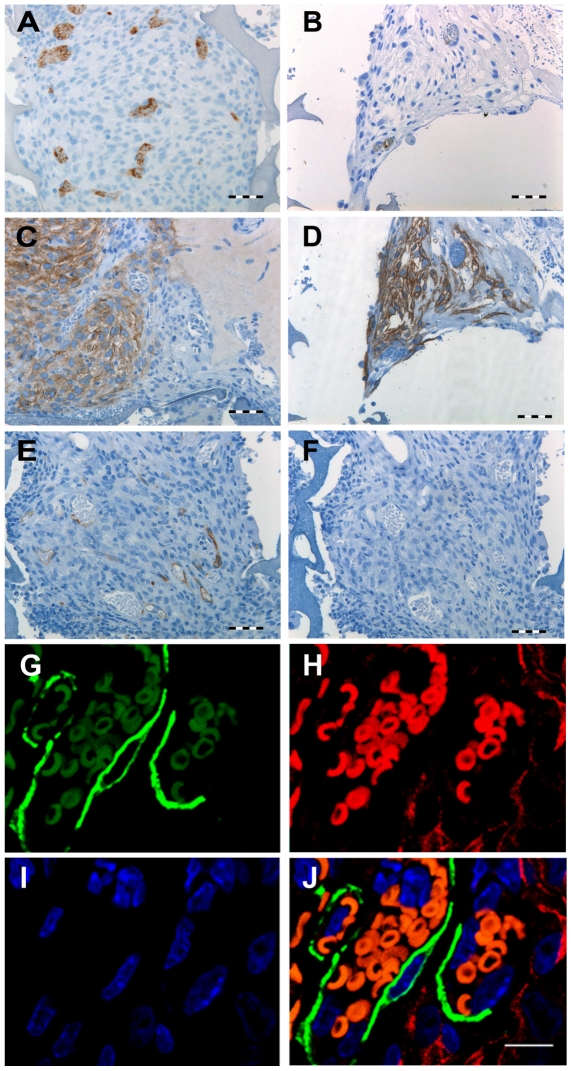
Matrigel encapsulated sponge angiogenesis assay (MESA). Consecutive 4 µm histological sections from matrigel plugs isolated after 7 days *in vivo*. *A*,*B*: Anti-FITC antibody visualized by brown chromogen diaminobenzidine, detected the blood pooling agent FITC-Dextran, indicating anastomosis with host circulation. *A*: Sponge region with -BD11 cells. *B*: Sponge region with -BC8 cells. *C*,*D*: Human specific anti-CD99 was used to confirm presence of *C*: -BD11 and *D*: -BC8 cells. *E*: Murine specific anti-CD34 stain of the sponge region with -BD11 cells. *F*: A parallel section stained with human specific anti-CD31. *G–J*: Laser scanning confocal microscopy of 4 µm histological sections of plugs seeded with -BD11 cells 63× magnification. *G*: α-smooth muscle actin stain visualized with goat anti mouse IgG2b Alexa 488 (green); *H*: TRA-1-85 stain visualized with goat anti mouse IgG1 Alexa 555 (red); *I*: DAPI stain of nuclei (blue); *J*: *G*–*I* overlay. N.B. red blood cells have red and green spectrum autofluorescence and appear orange. No cells double-stained for α-smooth muscle actin and TRA-1-85. Scale bar, *A*–*F*: 100 µm; *G*–*J*: 10 µm.

The matrigel encapsulated sponge angiogenesis (MESA) assay tested whether spontaneous *ex vivo* capillary morphogenesis correlated with neovascularization. The one-week angiogenic response was greater for -BD11 than -BC8 cells. Clone -BD11 MESA assays had numerous vessels containing a FITC positive lumen (Figure 2*A*), confirming functional conduit vessels, but this was rare in -BC8 assays (Figure 2*B*). Nonetheless in both cases, hMSC cells within the sponge scaffold were CD99+, a marker for human cells (Figure 2*C*, 2*D*). The staining pattern for endothelial cell biomarkers was positive for murine-specific CD34 (Figure 2*E*) and negative for human-specific CD31 (Figure 2*F*), thus endothelial cells were host derived. Most vascular mural cells strongly positive for α-smooth muscle actin did not co-stain with human specific biomarker TRA-1-85 (Figure 2*G*–2*J*). However, TRA-1-85+ cells were found directly adjacent to endothelial cells, suggesting a possibly mixed murine and human cell contribution to pericyte cells in early stages of vessel formation.

### Decellularized -BD11 matrix promoted human endothelial cell tubular morphogenesis *ex vivo*

**Figure 3:**
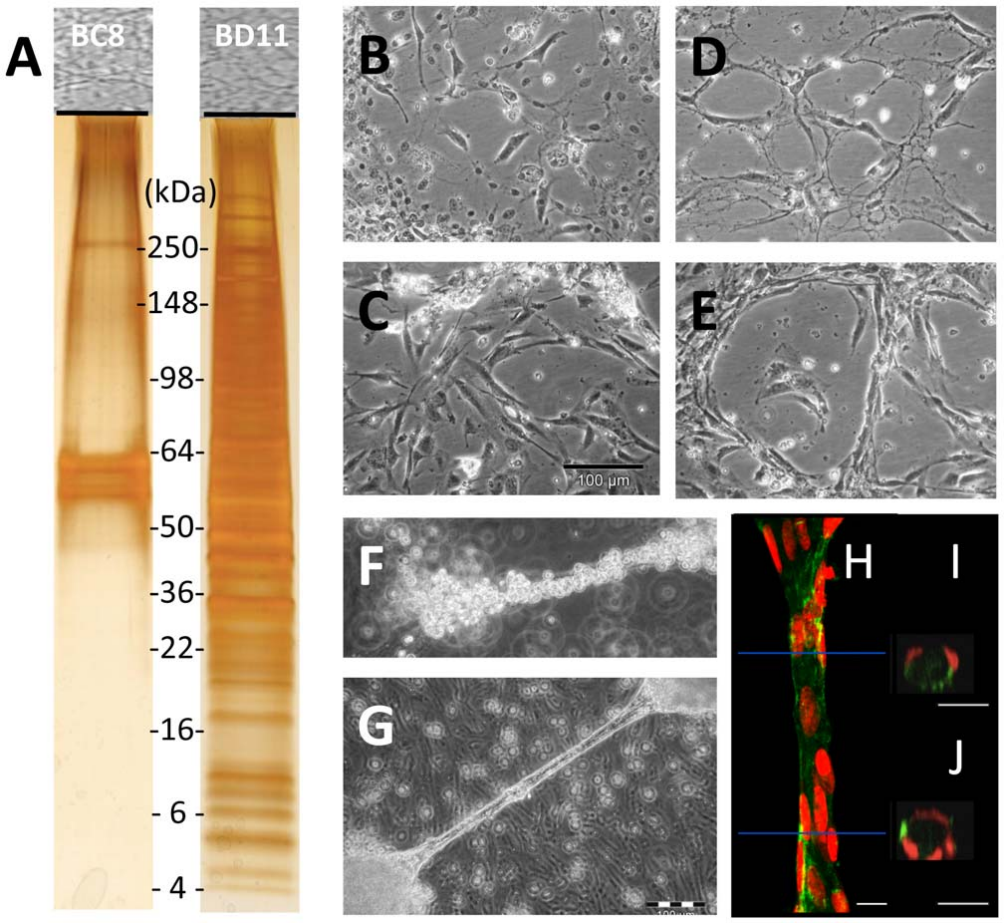
Analysis of hMSC-TERT-BC8 and -BD11 extracellular matrix (ECM). Sodium dodecyl sulfate polyacrylamide gel electrophoresis of equivalent total protein extracts of decellularized cells. *A*: Silver stained -BC8 proteins (left lane) and -BD11 proteins (right lane). Phase contrast photomicrographs of TIME endothelial cells seeded on decellularized matrix derived from *B*,*C*: -BC8 and *D*,*E* -BD11 clones on plastic dishes after *B*,*D*: 1 day or *C*,*E*: 10 days after seeding. Tubular cord formation by TIME cells when seeded on decellularized -BD11 matrix. *F*: Phase contrast photomicrographs of TIME cells aligned along detached free-floating cords of decellularized matrix within 2 hours of seeding. *G*: At 21 days, stable endothelial cord structures were maintained. *H–J*: 3D reconstructed images of TIME cells from a 21 day old cord structure stained with a FITC labeled Ulex Europaeus agglutinin lectin I (green) and propidium iodine counterstained nuclei (red) obtained with confocal microscopy. *H*: A longitudinal view of the tubule at 63× magnification. Red lines indicate region corresponding to XZ sections in adjacent figures. *I*,*J*: XZ-section stacks were used for cross section 3D-reconstruction showing TIME cell tube-like organization. Scale bar, *B–E*: 100 µm; *H*–*J*: 10 µm.

Since expression of angiogenic factors VEGF-A and Angiopoietin-1 were equivalent and unlikely to explain differences in the revascularization potential of the clone-derived tumors, we explored extracellular matrix (ECM) expression. Our results agreed with studies that emphasized a role for hMSC ECM in maintaining vasculature structure [17]. Despite similar cell morphology under phase contrast microscopy, protein gel electrophoresis of equivalent amounts of decellularized matrix from confluent -BC8 versus -BD11 monolayer cultures differed markedly, with a much more complex protein band pattern for clone -BD11 (Fig. 3*A*).

Comparing the endothelial support of decellularized matrix from serum-starved clones, TIME endothelial cells association with matrix from -BC8 cells only poorly, lacking close alignment (Figure 3*B*, 3*C*). In contrast, TIME cells closely populated the decellularized matrix from -BD11 cells forming an aligned cord-like network pattern (Figure 3*D*, *3E*). Consistent with previous observations [18] the -BD11 clone could form long cellular cords elevated above the surface of the monolayer, surrounded by culture medium. Suspended cords of matrix, retained after gentle decellularization, were templates for rapid adhesion and alignment of freshly seeded endothelial cells (Figure 3*F*). Subsequently, a stable endothelial cell tubular structure formed around the -BD11 matrix (Figure 3*G*) with aligned cell nuclei in cells circumscribing the matrix scaffold (Figures 3*H*–3*J*).

### Decellularized -BD11 matrix promoted endothelial cell migration *in vivo*

**Figure 4:**
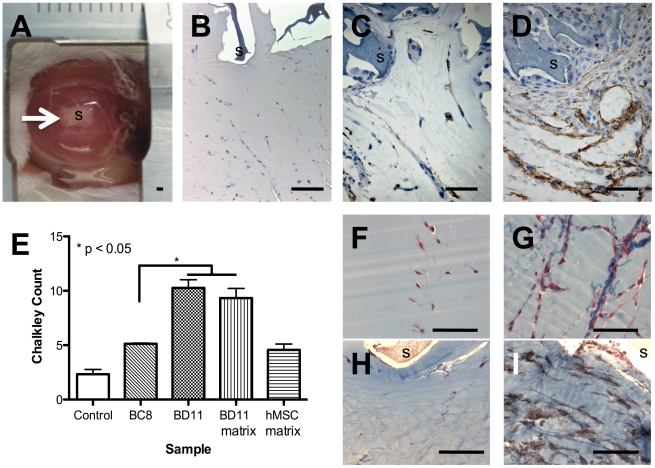
Histological sections of MESA plugs after 7 days *in vivo*. *A*: Photomicrograph of a MESA implant showing a 1 cm diameter matrigel plug with a centrally implanted sponge (arrow, “S”). *B*: Haemotoxylin and Eosin stain of a sponge loaded with ECBM-MV2 medium. *C*,*D*: Histological sections stained with murine specific anti-CD34 antibody visualized by brown chromogen diaminobenzidine. *C*: Field of view of matrigel adjacent to sponge seeded with -BC8 cells. *D*: Field of view of matrigel adjacent to sponge seeded with -BD11 cells. *E*: Chalkley count quantification of vasculature adjacent to the Matrigel® embedded sponge. *P<0.05, Kruskal-Wallis. *F*,*G*: Histological sections showing migratory cells within the Matrigel® surrounding *F*, control medium sponges or *G–I*: sponges with -BD11 decellularized matrix, stained blue with Masson's trichrome. *H*,*I*: Anti-CD34 antibody was used to visualize endothelial cells (brown) in regions of the Matrigel® *I*: with or *H*: without decellularized matrix. Scale bar, 100 µm.

In the sensitive directional MESA assay (Figure 4*A*), very few CD34+ murine endothelial cells migrated through the Matrigel® plug towards the control sponge pre-incubated with culture medium alone (Figure 4*B*). For sponges loaded with -BC8 cells, few murine CD34^+^ endothelial cells reached the sponge periphery (Figure 4*C*), but loaded with -BD11 cells many more did so (Figure 4*D*); Chalkley counts indicated a significant difference (Fig. 4*E*), p = 0.02 (Kruskal-Wallis). Given decellularized matrix-endothelial interactions *ex vivo*, the MESA assay was repeated using decellularized -BD11 matrix. In contrast to control MESA assays (Fig. 4*F*) sponges loaded with decellularized matrix contained closely aligned migratory endothelial cells attached to the blue stained matrix (Figure 4*G*). Within the same matrigel plug, regions without collagen fibril staining had very few endothelial cells (Figure 4*H*), whereas matrix-dense regions had numerous CD34^+^ murine endothelial cells (Figure 4*I*). The resulting mean Chalkley count for decellularized matrix in the MESA assay (9.3±1.53) was significantly greater than the control sponge (2.3±0.76), approaching the score for whole -BD11 cells (10.27±1.2). Decellularized matrix from primary hMSC led to Chalkley counts equivalent to clone -BC8 rather than -BD11 (Figure 4*E*), highlighting the latter clone had distinctive extracellular matrix with greater angiogenic potential than untransformed hMSC.

### SILAC Mass spectrometry of proteins in decellularized matrix from -BD11 cells

Since decellularized -BD11 matrix efficiently evoked excellent endothelial tubular structures *ex vivo* and promoted neovascularization, we aimed to identify inherent surface proteins that might account for endothelial attachment, activation and *in vivo* chemoattraction. Mass spectrometry peptide identification achieved high levels of confidence, with individual peptide ion identity having a false-positive rate estimation of <0.05. Favouring a protocol for endothelial recellularization [19], rather than for absolute matrix purity it was expected that we would co-purify intracellular proteins with the decellularized matrix. Given -BD11 cell sprouting, it was noteworthy that intracellular proteins included Protein kinase Cδ, IQmotif GTPase (IQGAP1), Ras associated protein (Rap1b), Chloride Intracellular Channel 4 (CLIC4), Cofilin-1, Fascin, Myosin 9, Profilin-1, Talin-1 and Vimentin. More relevant for interaction with endothelial cells, we identified SILAC-labelled peptides for 50 cell surface/extracellular proteins with angiogenic function (Table 1).

**Table 1:** Angiogenic ECM/cell surface proteins in -BD11 decellularized matrix.

Protein name	Accession number	Gene symbol	Cellular location§	Evidence for role in angiogenesis [Reference]
Activated leukocyte cell adhesion molecule (CD166)	IPI00015102.2	ALCAM	PM	Targeted Antibodies diminished endothelial capillary formation induced by Galectin-8 [83]
Aminopeptidase N (CD13)	IPI00221224.6	ANPEP	PM	Knockout mice show impaired angiogenesis [51]
CD44 Antigen precursor	IPI00305064.1	CD44	PM	Mediates activity of antiangiogenic peptide [57]
CD47	IPI00413696.5	CD47	PM	Thrombospondin-1 receptor, antagonises nitric oxide [84]
Enolase 1	IPI00465248.5	ENO1	PM	A hypoxia-induced protein in endothelial cells [85]
Epidermal Growth factor receptor	IPI00018274.1	EGFR	PM	Important for angiogenic factor secretion by MSC [86]
Glycoprotein non metastatic protein B	IPI00470529.3	GPNMB	PM	Shed ectodomain enhances endothelial recruitment [49]
Insulin Like Growth Factor 2 receptor	IPI00289819.4	IGF2R	PM	Mediates endothelial progenitor cell homing [87]
Integrin alpha-2 precursor	IPI00013744.1	ITGA2	PM	Mediates endothelial *ex vivo* capillary morphogenesis [88]
Integrin alpha-3 precursor (CD49c)	IPI00290043.1	ITGA3	PM	Alpha3beta1 integrin mediated inter-cell crosstalk for endothelial migration [89]
Integrin alpha-5 precursor	IPI00306604.5	ITGA5	PM	Targeting siRNA caused vessel shrinkage [90]
Integrin alpha-6 precursor (CD49f)	IPI00010697.1	ITGA6	PM	Increased expression in angiogenic tumour vessels [91]
Integrin alpha-V precursor (CD51)	IPI00027505.2	ITGAV	PM	An anti-angiogenic target [90]
Integrin beta 1 precursor	IPI00217563.3	ITGB1	PM	Gene inactivation via Cre-loxP in mice caused mural cell defects [92]
Integrin beta 3 precursor	IPI00303283.2	ITGB3	PM	Antibody reduced adhesion of MSC to endothelium [59]
Integrin beta 5 precursor	IPI00788112.1	ITGB5	PM	Overexpression enhanced angiogenic cell function [93]
Metadherin	IPI00328715.4	MTDH	PM	Mediates breast cancer cell adhesion to endothelium [94]
Neuropilin 1	IPI00398715.5	NRP1	PM	Receptor for angiogenic factors [95]
Nucleolin	IPI00444262.3	NCL	PM	Mediates cell migration and tubule formation in angiogenic vessels [96]
Platelet-derived growth factor receptor beta precursor	IPI00015902.3	PDGFRB	PM	Targeted antibodies prevented MSC tubule formation on matrigel [97]
Pro low-density lipoprotein receptor related protein 1 precursor (CD91)	IPI00020557.1	LRP1	PM	Regulates ECM in blood vessel formation and stabilization [98]
Semaphorin 7A	IPI00025257.1	SEMA7A	PM	Binds integrins, promotes axon outgrowth [95]
Talin-1	IPI00298994.5	TLN	PM	Maintains integrin complexes interacting with VCAM-1 [99]
Tetraspanin	IPI00298851.4	CD151	PM	Complexes with integrins to enhance angiogenesis [100]
Thy-1 cell surface antigen (CD90)	IPI00022892.2	THY1	PM	Cytomegalovirus induced down regulation led to vascular disease [101]
Annexin A2	IPI00455315.4	ANXA2	ECM	Interacts with pro-angiogenic S100A4 protein [102]
Basigin (CD147)	IPI00795150.1	BSG/EMMPRIN	ECM	Stimulates endothelial cell migration and tube formation [103]
Cathepsin S	IPI00299150.4	CTSS	ECM	Promotes pericellular hydrolysis and targeted antibodies inhibited angiogenesis [55]
collagen, type VI, alpha 1	IPI00291136.4	COL6A1	ECM	Associated with tumour stroma and matrix remodelling for microvasculature [104]
collagen, type VI, alpha 2 precursor	IPI00304840.4	COL6A2	ECM	ibid
collagen, type VI, alpha 3 precursor	IPI00022200.2	COL6A3	ECM	ibid
Elastin microfibril interfacer 1	IP100013079.1	EMILIN1	ECM	Knockout mice have vascular defects [105]
Fibronectin 1 isoform4 preprotein	IPI00414283.5	FN1	ECM	Promotes brain endothelial cell survival and growth [106]
Fibulin-1	IPI00218803.2	FBLN1	ECM	Binds angiogenin, stabilizes new blood vessel walls [107]
Galectin-1	IPI00219219.3	LGALS1	ECM	Essential for tumor angiogenesis [73]
Galectin-3 binding protein	IPI00023673.1	LGALS3BP	ECM	Implicated as an angiogenic factor from gene expression data mining [108]
Laminin B1	IPI00853454.1	LAMB1	ECM	Increases endothelial sprout formation [91]
Matrix Metalloproteinase-1	IPI00008561.1	MMP1	ECM	Inducible by hypoxia in human bone marrow MSC [109]
Matrix Metalloproteinase-3	IPI00027782.1	MMP3	ECM	Upregulated by hypoxia in MSC [109]
Matrix Metalloproteinase-14	IPI00218398.5	MMP14	ECM	Plays a critical role in MSC-mediated sprouting [56]
Perlecan	IPI00024284.4	PLC	ECM	Impaired angiogenesis in Perlecan deficient mice [110]
Tenascin C	IPI00220213.1	TNC	ECM	Mediator of postnatal cardiac angiogenesis [111]
Thrombospondin-1	IPI00296099.6	THBS1	ECM	Negative modulator of angiogenesis, activates latent TGF-ß1 [40]
Transforming growth factor beta induced	IPI00018219.1	TGFBI	ECM	Mediates lymphatic endothelial cell adhesion to ECM in low oxygen [112]
Transglutaminase 2	IPI00218251.1	TGM2	ECM	Autoantibodies disturb angiogenesis [113]
Versican isoform 1	IPI00009802.1	VCAN	ECM	Versican G3 domain promotes angiogenesis [114]
High Mobility Group Box 1	IPI00419258.4	HMGB1	S	An angiogenic switch molecule [50]
HtrA1 Serine Peptidase 1	IPI00003176.1	HTRA1	S	Mutated in single gene disorder of cerebral small vessels CARASIL [115]
Lactadherin	IPI00002236.3	MFGE8	S	Binds integrins with a crucial role in VEGF-mediated neovascularization [116]
Macrophage migrationinhibitory factor	IPI00293276.1	MIF	S	Chemotactic for endothelial progenitor cells [117]
Phosphoglycerate kinase I	IPI00169383.3	PGK1	S	Secretable glycolytic enzyme regulated by CXCR4 [118]
Wingless type MMTV integration site member 5A	IPI00013178.5	WNT5a	S	Regulates human endothelial cell proliferation and migration [119]

§ PM = Plasma Membrane, ECM = Extracellular Matrix, S = Secreted.

### Endothelial cell attachment to decellularized matrix *ex vivo* required -BD11 Galectin-1 expression

**Figure 5:**
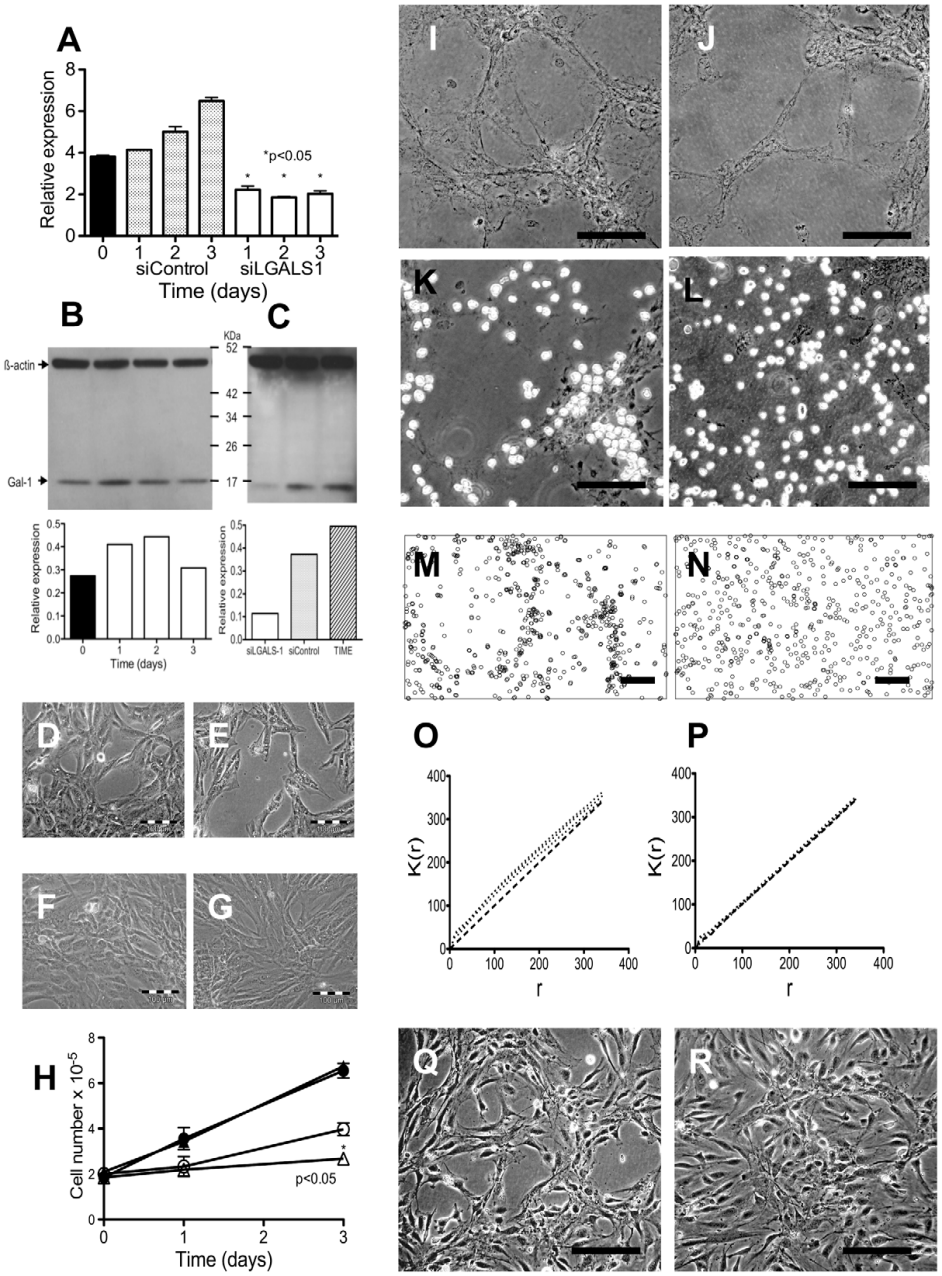
Endothelial cell attachment to decellularized matrix *ex vivo* required -BD11 Galectin-1 expression. *A*: RT-PCR analysis of LGALS1 gene expression in -BD11 cells grown in MEM with 10% FBS (day 0) versus serum starved cells treated with control siRNA (siControl) or anti-Galectin-1 siRNA (siLGALS1). *B*: Western blot of Galectin-1 protein expression in -BD11 cells grown in MEM with 10% FBS (day 0) versus cells serum starved for 3 days (clear bars). *C*: Western blot analysis of Galectin-1 protein in serum starved -BD11 cells 3 days after transfection with anti-LGALS1 siRNA (siLGALS-1) or control siRNA (siControl), versus routinely cultured TIME cells. *D–G*: Phase contrast photomicrograph of -BD11 cell monolayers grown *D,E*: with 10% FBS or *F,G*: without serum three days after treatment with *D,F*: control siRNA or *E,G*: anti-LGALS1 siRNA. *H*: Growth of -BD11 cells in 10% FBS (•,▴) or without FBS (○,▵) after transfection with control siRNA (○,•) or anti-LGALS1 siRNA (▵,▴). * p<0.05. I–L: Phase contrast photomicrograph of -BD11 decellularized matrix from 3 days serum-starved cultures of cells transfected with *I,K*: siControl or *J,L*: siLGALS1 *I,J*: before seeding with TIME endothelial cells and *K,L*: 30 minutes after seeding. *M,P*: ImageJ software rendition of endothelial cell distribution in 645.6 µm×433.5 µm fields used to determine spatial point coordinate data for endothelial cells seeded on matrix from -BD11 cells treated with *M*,: siControl or *N,P*: siGALS1. Ripley's K function graphs for Time endothelial cell distribution on decellularized matrix from -BD11 cells treated with *O*: siControl or *P*: siLGALS1. *Q,R*: Photomicrographs of Time cells 10 days after seeding on decellularized matrix from -BD11 cells treated with *Q*: siControl or *R*: siLGALS1. Scale bar, 100 µm.

Galectin-1 was abundantly expressed in primary bone marrow derived hMSC [20] and was chosen from the list of SILAC-labelled proteins as a target for functional analysis to test our cellular model. In comparison to the Galectin-1 levels of the initial -BD11 population in 10% FBS, serum starved -BD11 cells showed a ≈40% increase in Galectin-1 mRNA (Figure 5*A*) and protein expression (Figure 5*B*). The levels of Galectin-1 protein expression in -BD11 cell Western blots resembled that of TIME endothelial cells (Figure 5*C*). The siRNA mediated knock down of Galectin-1 expression was confirmed at the mRNA (Figure 5*A*) and protein (Figure 5*C*) level. In -BD11 cells grown in medium supplemented with 10% FBS, siRNA knock down of Galectin-1 had no marked effect on cell morphology (Figure 5*D*, 5*E*) or growth rate (Figure 5*H*). However, in serum-starved conditions, LGALS1 siRNA treated BD11 cells formed a less uniform network pattern (Figure 5*G*) and at 72 hours cell number was modestly reduced by ≈35% compared to BD11 cells transfected with sham control siRNA (Figure 5*H*). TIME human endothelial cells seeded on the decellularized matrix prepared from -BD11 cells transfected with sham siControl RNA and serum-starved for 72 hours (Figure 5*I*), attached preferentially to the underlying matrix rather than culture plastic; forming a corresponding pattern of aligned endothelial cells within 30 minutes (Figure 5*K*). In contrast, when using decellularized matrix prepared from siGALS1 knock down -BD11 cells (Figure 5*J*), the endothelial cells showed no preferential attachment to the matrix (Figure 5*L*). We tested whether attachment to the underlying matrix network significantly influenced the pattern of endothelial cell distribution, by determining the Ripley's K function for the seeded endothelial cells. ImageJ software determined spatial point coordinate data for endothelial cells seeded on matrix from -BD11 cells treated with siControl (Figure 5*M*) or siGALSN1 (Figure 5*N*). The observed point patterns of endothelial cells on siControl -BD11 decellularized matrix were consistently above a random expectation (Figure 5*O*) reflecting non-random covariate cell clustering on the matrix. Pattern intensity for endothelial cells seeded on siGALSN1 decellularized matrix led to curves matching an expected random Poisson pattern distribution (Figure 5*P*), reflecting that endothelial cells did not selectively attach to the underlying decellularized matrix. These differences in attachment to decellularized matrix were not only transient; different cell distribution patterns persisted for at least ten days in culture (Figure 5*Q*, 5*R*).

### Endothelial cell association with -BD11 cells *in vivo* required matrix Galectin-1 expression

**Figure 6:**
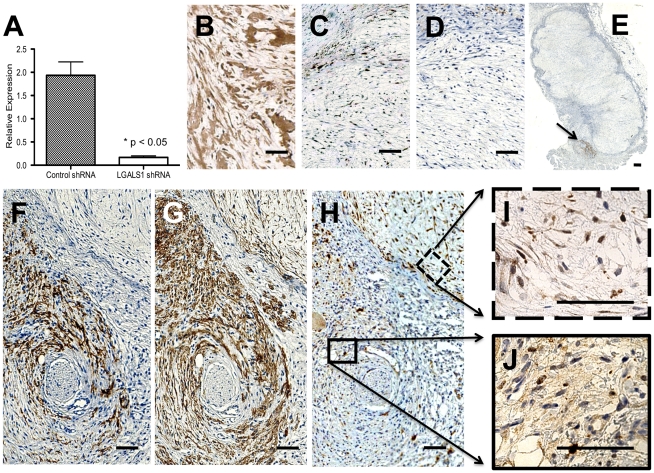
Endothelial cell association with -BD11 cells *in vivo* required matrix Galectin-1 expression. A: RT-PCR analysis of LGALS1 gene expression in pooled populations of -BD11 cells transfected with shRNA lentiviral vectors targeting a scrambled sequence (Control shRNA) or Galectin-1 (LGALS1 shRNA). B–J: Histomorphology of -BD11 transfectant tumour sections immunohistochemically stained (brown) for B: Galectin-1 in cells transfected with control shRNA or C: Galectin-1 in cells transfected with LGALS1 shRNA. D: CD34 immunohistochemical staining targeted murine endothelial cells in a parallel serial section equivalent to C. E: Whole tumour section of -BD11 cells transfected with LGALS1 shRNA with neighbouring subregion immunohistochemically stained for CD34 (arrow). F–H: Higher power magnification of arrow region in E, stained for F: CD34, G: Human-specific CD99, H: Galectin-1. Higher power magnification of Galectin-1 staining in regions in H that were I: CD99+/CD34− and J: CD99+/CD34+. Scale bar, 100 µm.

To more stringently test a role for Galectin-1 in -BD11-endothelial interaction we used shRNA vector technology to obtain pooled colonies of -BD11 cells with more stable knock down of Galectin-1 (Figure 6A). Tumours (n = 4) arising from -BD11 pooled cells transfected with shGALSN1 vector reached an average volume of 2.52 cm^3^ after 2 weeks, in close agreement with previous tumorigenicity studies [11]. Nonetheless, immunohistochemistry showed that unlike the multi-compartment intracellular and extracellular galectin-1 expression seen in shControl -BD11 tumours (Figure 6B), for -BD11 tumor cells transfected with the shGALSN1 vector, galectin-1 expression was restricted to the nucleus (Figure 6C). Though observed in the tumour periphery, serial sections showed remarkably few CD34+ murine endothelial cells amongst the CD99+ tumor cells, Chalkley counts typically <2±0.4 (Figure 6D). In one case, a dense cluster of CD34+ cells was found closely adjacent to the main tumour mass (Figure 6E, 6F) and this region colocalised with CD99+ human cells (Figure 6G). Immunohistochemical analysis of Galectin-1 (Figure 6H) revealed close association of CD34+ murine endothelial cells with human cells only where Galectin-1 expression was also prominent in the extracellular matrix (Figure 6I, 6J).

## Discussion

The heterogenous tumorigenic phenotypes among hMSC-TERT20 clones advantageously involved a cell type capable of contributing to vasculature as a pericyte. Given a rate-limiting influence of angiogenesis on tumorigenicity, we conjectured that clones with fast-growing tumours would express an optimal phenoype for acquisition of a blood supply. In agreement, the -BD11 clone expressed relatively high levels of α-smooth muscle actin and induced greater vascularity *in vivo*. Moreover, *ex vivo* survival of serum starvation included autonomous formation of stable cell cord networks, attributable to production of a more complex ECM [21]. Decellularized matrix prepared from serum-starved -BD11 cells induced endothelial cell cord formation *ex vivo* and angiogenesis *in vivo*. We used cell-labelled SILAC proteomics to identify 50 angiogenic proteins in the decellularized matrix with roles in endothelial chemoattraction, attachment and activation for sprouting and tube formation. Targeting galectin-1 revealed crucial roles in mediating both *ex vivo* serum-starved -BD11 matrix-human endothelial cell interactions and *in vivo* associations between these xenografted human hMSC and murine endothelial cells.

Our model may introduce biases specific for the angiogenic potency of telomerized hMSC within the context of tumour formation. However, global gene expression studies noted close overall similarity between disparate angiogenic situations and highlighted a role for ECM molecules [22]. Subregions of primary hMSC cultures sometimes showed autonomous cord morphogenesis in growth factor depleted conditions, supporting relevance for non-transformed bone marrow stromal cells. Spontaneous capillary morphogenesis on culture plastic under serum-free conditions, though rare, was also reported in a selected murine endothelial cell line, F-2C [23]. The -BD11 cells provide the first example of a human cell line displaying such a phenotype. A key advantage is that *ex vivo* cord formation studies no longer required Matrigel™ (a complex mixture of murine laminin, type IV collagen and fibronectin extracellular matrix components derived from murine Engelbroth-Holm-Swarm (EHS) sarcomas) as an inductive substrate. Its composition poorly represents the typical interstitial matrix microenvironment of endothelial cells during physiological angiogenesis *in vivo* [24], [25] and our experiments were not subject to batch variation. The -BD11 decellularized matrix provided an autonomous human serum-free microenvironment to better explore angiogenic responses to matrix components.

Serum deprivation was toxic for hMSC-TERT20-BC8 cells but surprisingly not for -BD11 cells. Primary human mesenchymal stem cells were susceptible to death after hypoxia but much more so when combined with serum starvation [26]. Mechanisms underlying the survival of the -BD11 cells will be the focus of future studies. An attractive hypothesis is that starved hMSC resist stress by adopting a “default” subsistence phenotype that encourages new vessel growth. The adaptive response to serum starvation may include expression of hypoxia-inducible mRNAs regulated by changes in translation efficiency [27]. Supporting this view, -BD11 cells underwent a 4-fold increase in translation protein eIF4G/eIF4E ratio when starved of serum, likely to reflect reduced 4E-BP levels [28]. Stable knockdown of 4E-BP1 can contribute to expression of proteins associated with cytoskeletal organization, invasion and hypoxia-regulated genes [29]. Elucidating such mechanisms has implications for both tumour biology and stem cell therapy, given that *ex vivo* preconditioning via hypoxia improved ischemic therapy with human mesenchymal stem cells [30].

Notably, among the genes expressed during -BD11 serum-free cord morphogenesis, were relatively low levels of CD31 and VEGFR-2, but Angiopoietin-1 (Ang-1) distinguished these cells from TIME microvascular endothelial cells, emphasizing a more pericyte than endothelial phenotype. In addition, gene expression for the Ang-1 receptor Tie-2 has been attributed to a mesenchymal subpopulation of pericyte progenitors [31]. Blood vessels of Tie-2 knockout mice lacked mural cells and a similar poor endothelial cell association with mesenchymal cells and surrounding matrix was seen in Ang-1 knockouts. The manner in which Ang-1 is presented to the endothelial cells has an important influence on subsequent Tie-2 mediated signalling pathways. For matrix-bound Ang-1, endothelial Tie-2 signalling regulated migration through Erk signaling pathways, but associated with cell-cell interactions, Tie-2 bridged adjacent cell junctions and Ang-1 preferentially activated endothelial Akt signaling, stimulating vascular quiescence. Thus, Ang-1 could modulate endothelial cell sprouting and control vascular quiescence and stabilization [32] [33]. Consistent with expression of pericyte genes, -BD11 cells were histologically located adjacent to endothelial cells *in vivo*.

Cell clone differences governing cell survival may overshadow those governing angiogenic potency and complicate data interpretation. Thus we focused on the most angiogenic clone for proteomic characterisation to account for -BD11 cord network formation and interaction with endothelial cells. SILAC proteome analysis selectively detected -BD11 synthesized proteins, overcoming the problem of artefactual identifications from any contaminant serum proteins. We identified intracellular proteins likely to be involved in -BD11 cord formation and cell surface molecules likely to mediate chemoattractive recruitment, cell-cell interactions and activation of endothelial cells. Among SILAC-labelled intracellular proteins co-purified with the -BD11 decellularized matrix, PKCδ could be induced by serum deprivation and activated cell scattering [34]. Angiogenic GTPase signaling molecules, such as IQGAP1 could interact with VEGFR-2 [35] and Rap1b, an integrin activating molecule, could induce angiogenic sprouting [36]. Chloride Intracellular Channel 4 (CLIC4) was also implicated in early stages of endothelial tubular morphogenesis by proteomic studies [37]. The autonomous -BD11 cell sprouting upon serum starvation challenged traditional “endothelial first” angiogenic models whereby pericytes are recruited by migrating endothelial cells that lead the tubulogenic process. Our *ex vivo* response of endothelial cells to -BD11 decellularized matrix was consistent with *in vivo* observations that it is pericytes that initiate sprouting by forming strands connected to existing capillaries and endothelial cells use these “cellular cables” as guidance cues during their movement to complete vessel assembly [38]. Others have also noted that pericytes can bridge gaps between the leading edges of opposite endothelial sprouts, implicating they may serve as guiding structures for outgrowing endothelial cells [39].

An early role for pericytes would be advantageous for therapeutic application and our finding that hMSC decellularized matrix *per se* effectively enhanced neoangiogenesis *in vivo* was very encouraging. This was not a foregone conclusion, since both positive and negative interactions balance vascular ECM morphogenesis or regression and some stromal cell types induced apoptosis when they interacted with endothelial cells [40]. The host remodeling response is sensitive to matrix preparation [41]; chemically crosslinked matrix scaffolds can resist degradation, inducing fibrous encapsulation and chronic inflammation rather than constructive remodeling. We did not attempt to retain tertiary structure when harvesting the decellularized matrix, given that degradation products of matrix bioscaffolds sufficed as modulators of recruitment and proliferation of endothelial cells [42]. Nor did we explore whether -BD11 decellularized matrix sequestered potent angiogenic factors such as VEGF and FGF-2, but for Sorrell *et al.* these cytokines did not explain the different angiogenic potency of ECM from different human dermal fibroblast subpopulations [43].

Surprisingly, we did not detect collagen type I in the decellularized matrix extracts [44] though we have independent evidence -BD11 cells secreted this collagen (data not shown). It is possible that in -BD11 cord morphogenesis collagen-I is expressed at relatively low levels. Soucy and Romer [45] noted that the compact arrangement of tenascin-C and collagen-VI filled more volume than collagen-I and endothelial cell matrix adhesions selectively targeted fibronectin. We detected tenascin-C, fibronectin and all three monomer chains of collagen-VI required for the triple helix structure that defines the locus of endothelial cell interaction [46]. Of special relevance, collagen-VI differed from collagen-I by being able to prevent apoptosis and allow proliferation of mesenchymal cells under serum-starved conditions [47].

Qualities attributed to decellularized matrix in a therapeutic engineered airway included a contribution to revascularization [48]. The -BD11 decellularized matrix compared well to whole cells with regard to neoangiogenic potency in the MESA assay. How could a cell attachment scaffold provide a near-equivalent response to whole cells that can also synthesize and secrete angiogenic chemokines? The proteomic characterization provided a more understandable view, with molecules that sequester growth factors and remodel matrix to dynamically govern the recruitment of endothelial cells, their interaction and activation to form tubular structures. The -BD11 decellularized matrix contained GPNMB, which shed from the cell surface by the matrix metalloproteinase ADAM10 enhanced recruitment of endothelial cells [49]. SILAC labelled ADAM10 was found in -BD11 supernatant (data not shown). The secreted form of HMGB1 has been shown to be sequesterable in ECM, able to recruit endothelial cells and stimulate sprouting [50]. APN is a membrane bound zinc-binding protease that participates in extracellular proteolysis with context dependent function. Though not essential for survival or physiological vascularization, APN-null mice showed a severely impaired angiogenic response to pathological conditions [51]. MMP1, MMP3 and MMP14 functions are not confined to the degradation of ECM components, but include activation of latent cytokines, cleaving membrane-anchored proteins and release of matrix-bound growth factors, generating bioactive neopeptides. The membrane-type family member MMP14, also known as MT1-MMP is one of the most influential metalloproteinases in the angiongenic process [52]. Cathepsins can cooperate with MMPs [53] and Cathepsin-S, enriched in sprouting tip cells [54] is required for angiogenesis [55]. In addition to specific mesenchymal cell proteolytic mechanisms [56], paracrine proteases from endothelial cells and local inflammatory cells also remodel the ECM during the vascular response, releasing chemokines [6]. CD44 is a cell surface proteoglycan that serves as a cognate receptor for MMP-9 and targeting the CD44 pathway inhibited endothelial migration and tubule formation more than endothelial proliferation [57].

The contribution of transmembrane α and ß heterodimer integrins (the most important receptor family mediating cell adhesion to ECM) to endothelial-pericyte interactions has been extensively reviewed [58]. The integrin subunits expressed by the -BD11 cells were in broad agreement with those described for primary MSC-endothelial cell interactions [59]. We also found modulators of integrin function, such as integrin ß1 binding semaphorin 7A [60] and Talin-1, a focal adhesion complex protein that regulates integrin interactions and controls pericyte contractility [61]. The stress response to serum starvation or hypoxia has been shown to modify integrin expression and function to favor ECM-cell interactions [62].

Regarding molecules that can sequester angiogenic cytokines, neuropilin-1 is a co-receptor for VEGF_165_ that can also bind Galectin-1 resulting in enhanced VEGFR-2 phosphorylation, to mediate migration and adhesion in endothelial cells [63]. Perlecan can bind many growth factors including BMP-2, CTGF, PDGF, FGF-2, nidogen1, nidogen2, α-dystroglycan and VEGF. It may create stable “signalosomes” by clustering transmembrane proteins and stabilizing their interactions. It also interacts with the α2ß1 cell surface integrin forming additional complexes linking ECM with the cell. The outcome of perlecan antisense targeting is context dependent; in human colon cancer xenografts it decreased neovascularization and tumor progression, whereas, in fibrosarcoma cells, the phenotype became more aggressive with increased migration and invasion [64].

Consistent with the long held view that there is dynamic reciprocity between matrix composition and gene expression [65], a number of SILAC labelled ECM proteins are also found intracellularly [66]. Dual localization proteins include EGFR, HMGB1, PDGFR, LGALS1, Nucleolin, ANXA2, TGM2 and MIF. Location can influence function; extracellular TGM2 can have a role in cell adhesion, whilst intracellular TGM2 can regulate apoptosis [67]. Confirming a cell surface role for nucleolin, blocking antibodies could suppress angiogenesis [68]. Our proteomic analysis did not fully resolve cellular location, emphasizing need for functional studies.

A protein with an important angiogenic role might be expected to persist or even have increased expression during starvation stress. Galectin-1, a highly expressed protein in primary hMSC, was strongly implicated in ECM-cell interactions [20]. Correlating with our serum-starved situation, in hypoxic stress conditions, fibroblasts expressed increased levels of Galectin-1 [69]. Whether Galectin-1 promotes or inhibits cell growth is context dependent [70]. Notably, -BD11 cells became more dependent on Galectin-1 for optimal growth when starved. Though siLGALS1 treated -BD11 cells retained an initial cord-morphogenesis response to serum starvation, qualities of the ECM were altered and subsequent preferential attachment of endothelial cells to the decellularized matrix was lost. Thus, Galectin-1 expression in the hMSC maintained a role modulating ECM-heterotypic cell interactions [20],[71]. Given these results, we used lentiviral vector shRNA GALSN1 transfection for more stable knock down to explore whether Galectin-1 also served as an effective tumour target in our -BD11 model. Surprisingly, -BD11 tumour growth from pooled colonies of transfected cells was not markedly affected despite a greatly reduced recruitment of host endothelial cells to the tumour mass. Heterogeneity in the microvascular density of -BD11 tumours was previously reported [12] and sarcomas may develop alternative means of circulation [5]. Histological analysis did detect nuclear Galectin-1 in the tumour cells, a phenotype similar to the persistence of nuclear nucleolin expression in the presence of its inhibitors of transcription and translation [72], but cell surface matrix expression of Galectin-1 was below detection. Perhaps arising from use of a heterogenous pool of transfected -BD11 cells we did observe a small exceptional region where the human cells expressed Galectin-1 in the matrix. Murine endothelial cells populated this region densely, confirming an *in vivo* requirement for -BD11 surface galectin-1 expression for endothelial interaction.

Recent studies have verified that Galectin-1 [73], [74] and other proteins identified in -BD11 ECM, e.g. aminopeptidase-N [75] annexin-A2 [76] or nucleolin [77], can serve as tumour targets, with evidence that combined targeting of perivascular and endothelial cells can enhance anti-tumour treatment [78]. Models that help clarify the ECM biology of hMSC will have significant implications for understanding the regulation of key angiogenic processes in tumorigenesis and ischemia. In a broader context, extracellular matrix bioactive peptides may have direct therapeutic application [79]. Decellularized matrix arguably provides lower clinical risks regarding immune rejection and tumorigenicity compared to whole cells. Yet biological effectiveness can vary greatly between similar sources or with just single gene alterations. Detailed understanding of decellularized matrix and its components is required for improved therapeutic application.

## Materials and Methods

### Cell culture

The hMSC-TERT20 clones, designated hMSC-TERT20-BC8 and hMSC-TERT20-BD11 and TIME cells were derived and cultured as described [11] in MEM (Gibco Invitrogen Co., Tastrup, Denmark) supplemented with 10% fetal bovine serum (FBS; Gibco Invitrogen Co., batch tested) or supplemented ECBM-MV2 medium (PromoCell GmbH, Heidelberg, Germany), respectively.

### Serum Starvation

hMSC-TERT20-BC8 and -BD11 cells were routinely cultured to 90% confluence in 6-well plates. After three washes with PBS^++^ (Gibco Invitrogen) cells were fed serum-free MEM (Gibco Invitrogen Co., Tastrup, Denmark) and re-fed every 3 days. At indicated time points, RNA was harvested for RT-PCR gene expression analysis, or cells were pelleted for Western blot analysis, fixed in 4% formaldehyde for immunocytochemistry, or detached with trypsin for FACS analysis.

### RNA extraction and reverse-transcriptase polymerase-chain reaction (RT-PCR)

Total RNA isolated from cultured cells used a single-step Trizol® (Invitrogen Co., Tastrup, Denmark) method, following manufacturer's instructions. RT-PCR used 20 pmole of forward and reverse primers (Table 2) as described [15]. PCR products were analyzed by 1.5% agarose gel electrophoresis, visualized with ethidium-bromide, and photographed.

**Table 2:** Primers used for RT-PCR.

Gene	Forward primer	Reverse primer	Annealing temp. (°C)	Amplicon size (bp)
Ang-1	5′-GCCATTACCAGTCAGAGGCAG-3′	5′-AATAGGCTCGGTTCCCTTCC-3′	60	70
Ang-2	5′-CGCTCGAATACGATGACTCG-3′	5′-CCACTGAGTGTTGTTTTCCATGAT-3′	60	72
Tie-1	5′-ACTTCACTTACGCGGGCATT-3′	5′-GCCACGTTCTGGCTGGAT-3′	60	68
Tie-2	5′-GGCAACTTGACTTCGGTGCT-3′	5′-GGCCTTGGTGTTGACTCTAGCT-3′	60	80
CD105	5′-CGCACCGATCCAGACCACTC-3′	5′-CCCGGCTCGATGGTGTTGGA-3′	60	188
CD31	5′-AAGGTCAGCAGCATCGTGG-3′	5′-AGTGCAGATATACGTCCC-3′	60	224
VEGF-A	5′-CTACCTCCACCATGCCAAGTG-3′	5′-TGATTCTGCCCTCCTCCTTCT-3′	55	62
VEGFR-2	5′-TGCCACCTCCATGTTTGATG-3′	5′-CAGCTGGAATGGCAGAAACTG-3′	60	188
LGalS1	5′-GGGTGGAGTCTTCTGACAGC-3′	5′-CTTGCTGTTGCACACGATG-3′	60	250
ß-actin	5′-TGTGCCCATCTACGAGGGGTATGC-3′	5′-GGTACATGGTGGTGCCGCCAGACA-3′	60	430

### Western blot analysis

At indicated time points, cells were washed twice with PBS containing Complete® protease inhibitor cocktail (Roche), scraped as whole cells and spun at 250 g for 15 minutes at 4°C before snap-freezing and storage at −180°C. Samples were resuspended in 100–200 µL lysis buffer (50 mM Tris/HCl, pH 7.5, 1 mM Na_4_P_2_O_7_, 1% Triton X-100, 2 mM EGTA, 2 mM EDTA-Na). Lysates sheared through a 26-guage needle, had total protein quantified by DC protein assay (Biorad, Munich, Germany). Equivalent samples (10 µg protein) and SeeBlue Plus2 pre-stained molecular size markers (Invitrogen) were loaded onto 4–12% Bis-Tris NuPage gels (Invitrogen) and run under denaturing conditions in NuPage MOPS-SDS buffer (Invitrogen). Proteins transferred to Invitrolon-PVDF filters in NuPage transfer buffer overnight at 4°C were incubated with blocking buffer SEA BLOCK (Pierce) for 1 hour at 4°C. Primary antibodies were applied for 1–3 days at 4°C, then secondary antibodies for 1 hour at 21°C. Immunoreactive proteins were detected with Q-dot Western blot reagent kits (Invitrogen) and ECL Plus Western blotting reagents (GE Life Sciences) per manufacturers' instructions and exposed to Hyperfilm ECL (GE Life Sciences). The diluted antibodies were mouse anti-human CD146 (NCL-CD126; Novocastra) at 1∶250, rabbit anti-human beta-actin (4967, Cell Signaling) at 1∶1000, rabbit anti-human profilin (IG706, Immunoglobe) at 1∶1000, mouse anti-human beta-actin (6276, Abcam) at 1∶5000, anti-mouse IgG1-HRP (7076, Cell Signaling) at 1∶2000 and anti-rabbit IgG-HRP (7074, cell Signaling) at a 1∶2000.

### *In vivo* Matrigel Encapsulated Sponge Angiogenesis (MESA) assay

A polyvinyl alcohol (PVA) 2 mm^3^ sponge (PVA unlimited, Warsaw, IN, USA) seeded with 1×10^5^ cells in serum supplemented medium was incubated overnight at 37°C, 5% CO_2_ in a 96-well ultra-low adhesion plate (Corning). Alternatively, sponges were cultured in medium alone, or centrifuged with decellularized matrix from 1×10^6^ cells serum-starved for 24 hours. Adopting a previous assay [80] and following institutional guidelines, 600 µl of cold growth factor reduced Matrigel (BD biosciences) was injected subcutaneously in 8-week old NOD/SCID mice to solidify within 20 minutes at body temperature. Needle orientation parallel to the tail improved plug geometry and uniformity. After a small skin incision, the treated PVA sponge was implanted centrally in the matrigel plug. After skin suture, mice were kept in an environment–controlled facility. At 7 days, 2.5 mg FITC-Dextran (Sigma) in 200 µL saline was injected in the tail vein, three minutes before sacrifice. The extracted matrigel/sponge pocket was fixed in 4% buffered formaldehyde for 24 hours at 4°C. Paraffin sections (4 µm), were stained with haematoxylin/eosin and antibodies for human specific CD31, clone JC/70A at 1∶50 dilution (Dakocytomation, Denmark), α-Smooth Muscle Actin, clone 1A4 at 1∶200 dilution (Dakocytomation, Denmark), human specific CD99, clone 12E7 at 1∶100 dilution (Dakocytomation, Denmark), mouse specific CD34, clone MEC14.7 at 1∶200 (Abcam ab8158) and Rabbit anti-FITC, polyclonal antibody at 1∶200 dilution (Dakocytomation, Denmark). Masson's trichrome stained matrix collagen fibres blue in samples using decellularized matrix. Murine microvessel quantification [12] used a 25-dot Chalkley microscope eyepiece graticule aligned with the sponge edge at ×200 magnification.

### Culture of endothelial cells on decellularized hMSC matrix

Clones hMSC-TERT20-BD11 and -BC8 were seeded at 10.000 cells/cm^2^ in 6 well plates in standard medium. After overnight attachment, cells were washed twice with PBS (Sigma/Gibco) before changing to serum-free MEM containing 1% P/S (Invitrogen). After 3 days of serum starvation, the monolayer placed on ice was washed in ice cold PBS^−−^ containing EDTA^+^ Complete® protease inhibitors (Roche). The cells were decellularized [19] for 3–5 minutes with 0.25% Triton X (Fluka), 0.25% sodium-deoxycholate (Merck) in PBS^−−^ (Gibco), and the isolated ECM was gently washed in PBS^++^(Gibco) with 100 µg/mL RNAse A (Roche) and 10 IU/mL DNAse (Sigma) followed by three washes in PBS^++^. Morphological changes were observed under phase-contrast illumination using an inverted microscope (Olympus) connected to a digital camera (Olympus, Denmark). Visual inspection ensured derivation of extracellular matrix without Hoechst dye-stained intact nuclei. For three independent hMSC matrix preparations, TIME cells (10.000 cells/cm^2^) were seeded on the matrix and the distribution of bright round refractive freshly attached cells was photographed after 30 minutes. Images were processed using ImageJ software to analyse a fixed window size of 645.6 µm×433.5 µm with thresholds isolating bright cells with points defined by intensity maxima. Ripley's K(t) function; K (t) = λ−1 E [ number of extra Events within distance t of a randomly chosen event], where λ is the intensity (number per unit area) was used to statistically summarize the point pattern, testing the hypothesis that under control conditions the endothelial distribution was not random, but covariant with the underlying decellularized matrix pattern.

### Mass spectrometry evaluation of hMSC-derived decellularized matrix proteins

#### In-Solution Digest

Decellularized matrix from hMSC-TERT20-BD11 cells was solubilized in 6 M urea/2 M thiourea (pH 8.0), 10 mM Tris pH 8.0. Proteins were reduced in 1 mM DDT (Sigma) for 45 minutes at room temperature and S-carbamidimethylated in 5.5 mM iodoacetamide (Sigma) in 50 mM NH_4_HCO_3_ for 30 minutes in the dark. Overnight protein digestion with 1 µg LysC at room temperature was followed by 4× dilution in 50 mM NH_4_HCO_3_ and addition of 1 µg trypsin (Sequencing grade, Promega) for overnight digestion at room temperature. The peptides were acidified with 3% (final concentration) trifluoracetic acid, then desalted and concentrated on C18 reverse-phase material micro-columns (Empore Disc, 3 M) [81].

#### Fourier transform mass spectrometry

A 7-Tesla LTQ-TF instrument (Thermo Fisher) coupled to an Agilent 1100 nanoflow liquid chromatography (LC) system (Agilent Technologies) provided LC tandem mass spectrometry (LC-MS/MS). The LC reverse-phase column was packed with ReproSil-Pur 120 C18-AQ 3 µm resin (Dr Maisch, GmbH). The mass spectrometer was operated in data dependent acquisition mode, the three most intense spectrum ions from selected ion monitoring (SIM) scans were chosen for accurate mass measurement.

#### Data analysis

Protein identification was via the MASCOT Search Engine (Matrix science). The major search criteria were as follows; Database: MSIPIslim_human (68992 sequences) [82]. Enzyme: MSIPI_DPTrypsin, allowing two missed cleavages. Fixed modification: Carbamidomethyl (C). Variable modifications: Acetyl (Protein N-term), Ammonia-loss (N-term C), Gln→pyro-Glu (N-term Q), Glu→pyro-Glu (N-term E), Oxidation (M), and Oxidaiton (P). MS/MS tol.: 0.8 Da. Peptide tol.: +/−2 ppm. The MSQuant v. 1.4.3a31 program (open source www.msquant.sourceforge.net) was used to set filters and manually validate protein identification. Identification criteria were: peptide length: at least 7 amino acids, at least two unique peptides with a Mascot score ≥25. Protein lists and additional GO information was collected using ProteinCenter software (Proxeon Biosystems A/S, Odense, Denmark).

### Immunofluorescent staining and Confocal Microscopy

Cells grown on glass chamber slides (Nunc, Denmark) were fixed in buffered 4% paraformaldehyde for 10 minutes at room temperature, washed ×3 with PBS (Sigma Aldrich) and incubated for 1 hour with primary antibodies in ChemMate Antibody diluent (Dakocytomation, Denmark). Human specific TRA-1-85 antibody (Chemicon) was diluted 1∶300, Anti α-smooth muscle actin, clone 1A4 (Dakocytomation, Denmark) was diluted 1∶200. Non-specific FC receptors were blocked with goat serum (Zymed, San Francisco, California, US) for 30 minutes. Slides were washed ×3 in PBS and incubated for 1 hour with compatible secondary antibodies (ALEXA Flour 555 and 488 Molecular Probes, USA), before mounting in DAPI medium (Dakocytomation, Denmark).

A Zeiss LSM 510 META confocal laser-scanning microscope obtained images with a 63×/1.2 W corr objective used an argon laser (488 nm) for excitation of Alexa 488, HeNe laser 543 nm for excitation of Cy3 and Alexa 555 and a two-photon (MaiTi XF-W2S) laser at wavelength 780 nm for excitation of DAPI. Pinholes for the HeNe laser and Argon laser were set to 1. Images were processed using NIH ImageJ 1,37c (http://rsb.info.nih.gov/ij/). The z-project feature and Aling3-TP plug-in for ImageJ made 3D reconstruction ortho images from 0.49 µm interval z-stacks.

### Transient siRNA Targeting of Galectin-1

ON-TARGETplus SMARTpool siRNA (Thermo Scientific) containing a mixture of four SMARTselection-designed siRNAs targeted the human Galectin-1 gene, LGALS1. The sense sequences of anti-LGALS1 siRNA were: 5′-CUAAGAGCUUCGUGCUGAA-3′; 5′-ACGGUGACUUCAAGAUCAA-3; 5-CCAGCAACCUGAAUCUCAA-3′; 5′-GCUGCCAGAUGGAUACGAA-3′. Corresponding scrambled siRNA served as control and the same short strands of siRNA coupled with fluorescein were used to confirm successful transfection. The final concentration of the siRNA duplex stock solution, dissolved in Dharmacon 5× siRNA buffer was 20 µM. Clone hMSC-TERT20-BD11 cells were reverse-transfected by seeding 2×10^5^ cells onto siRNA∶Lipofectamine 2000 (Invitrogen) complexes according to manufacturer's instructions. After overnight incubation the medium was changed to antibiotic and serum-free MEM. The mRNA and protein expression levels of Galectin-1 were evaluated during the first 3 days post-transfection. Cell number was counted from triplicate wells using a Nucleocounter (Chemometec A/S) per manufacturer's instructions. Decellularized matrix from siRNA transfected hMSC-TERT20-BD11 cells was prepared as described above, for testing interaction with newly-seeded TIME endothelial cells.

### Stable shRNA Targeting of Galectin-1 with lentiviral transfection

#### Cloning shRNA into Lentiviral Vector: Oligo Sequence

The following oligos were cloned into the pSicoR PGK puro vector (PMID: 15240889, Addgene): Non-targeting/scrambled, 5′TGAAGGCCAGACGCGAATTATTCAAGAGATAATTCGCGTCTGGCCTTCTTTTTTC-3′ sense, 5′TCGAGAAAAAAGAAGGCCAGACGCGAATTATCTCTTGAATAATTCGCGTCTGGCCTTCA-3′ antisense; target sequence GAAGGCCAGACGCGAATTA. LGALS1, 5′ TGCTGCCAGATGGATACGAATTCAAGAGATTCGTATCCATCTGGCAGCTTTTTTC-3′ sense, 5′ TCGAGAAAAAAGCTGCCAGATGGATACGAATCTCTTGAATTCGTATCCATCTGGCAGCA-3′ antisense; target sequence GCTGCCAGATGGATACGAA (PMID: 18431251).

#### Oligo Annealing, 5′phosphorlation

Oligos were first annealed by adding 1 nmol of sense plus antisense oligo, to 5 µL 10× annealing buffer (1 M Tris-HCl (pH 7.5), 5 M NaCl, 0.5 M EDTA) adding water to reach a total volume of 50 µL. The solution was incubated at 95°C for 4 minutes, 70°C for 10 minutes and cooled slowly to 20°C. For 5′phosphorlation, 100 pmol annealed Oligos was added to 2 µL 10× T4 ligase buffer (Promega) and 10 units of T4 Polynucleotide Kinase (Promega) with water to a total volume of 20 µL. The solution was then incubated at 37°C for 30 minutes and 70°C for 10 minutes.

#### Digestion, dephosphorylation of vector and ligation

1 µg of pSicoR PGK puro vector was digested with XhoI and HpaI (Promega) then 1 unit of TSAP (Thermosensitive Alkaline Phosphatase, Promega) was added. The solution was incubated at 37°C for 15–30 minutes and TSAP was heat inactivated at 74°C for 15 minutes. Ligation was done by adding 50 ng of pSicoR PGK puro vector to 0.25 pmol annealed and 5′phosphorylated oligo, 5 µL 2× Rapid LigBuffer (Promega) and 3 units of T4 ligase (Promega) with ddH2O added to a total volume of 10 µL. The solution was incubated at room temperature for 30 minutes and transformed into DH5α cells. Positive clones were analyzed by purifying the vector, using Wizard® Plus SV Minipreps DNA Purification System (Promega) according to manufactures instruction, analyzing linearized vector on a 0.5% agarose gel for 90 minutes at 70 V. Finally the insertion of correct insert into the vector was confirmed by sequencing.

#### Virus Generation and Infection

HEK293T cells (70–80% confluent, Genehunter) cultured in 6 well plates were transfected with 0.625 µg/well pMD2.G (Addgene) 1.25 µg/well psPAX2 (Addgene) and 1 µg/well pSicoR PGK puro constructs, either containing the LGALS1 oligo or the Non-targeting/scrambled oligo by using the FuGENE 6 (Roche) method according to manufacturers instruction. The supernatants, from 25 cm^2^ of HEK293T cells containing virus particles, were collected 24 and 48 h after transfection, filtered with a 0.45 µm filter, diluted 1∶1 with the culture medium, and added to hMSC-BD11 cells in a 25 cm^2^ flask supplemented with 6 g/mL Polybrene for infection. Twenty-four hours after a second round of infection, 3 g/mL puromycin was added for selection until all control cells were killed. The puromycin resistant cells were expanded and maintained in medium supplemented with 0.2 g/mL puromycin. An estimated 500.000 cells initially survived the selection to make the BD11-shLGALS1 and BD11-shControl pooled populations expanded and used for tumorigenicity studies within four passages of adenoviral vector transduction.

### Xenograft tumorigenicity

Immunodeficient mice (NOD/LtSz-Prkdcscid) were maintained in pathogen-free conditions. Cells (5×10^6^) below passage 5 were mixed with Matrigel 1∶1 before implantation (100 µL) to facilitate establishment and transplanted subcutaneously into the dorsal surface of 8-week old female NOD/SCID mice. After 14 days tumours were harvested and perpendicular diameters measured for an estimation of tumour volume. Tissue samples were fixed in 4% formaldehyde-0.075 mol/L NaPO4 (pH 7), dehydrated, embedded in paraffin and sectioned at 4 µm for histological and immunohistochemical evalulation.

### Immunohistochemistry of lentivirus transfected -BD11 cell tumours

For histological analysis, deparaffinised 4 µm thick sections were immunohistochemically stained with immunoperoxidase detection and Envision Plus according to manufacturer's instructions (Dako, Glostrup, Denmark). Murine specific anti-CD34 antibody and human specific anti-CD99 antibody were used as described above. Human specific anti-galectin-1 antibody clone 25C1 (Novocastra, Leica Biosystems, Newcastle, UK) was used at 1∶100 dilution. Haematoxylin and Eosin Y (Bie & Berntsens Reagenslaboratorium) was used as counterstain, photomicrographs were captured under bright field illumination with an inverted microscope DM4500 B equipped with Leica DFC300 FX Digital Color Camera (Leica Microsystems A/S, Herlev, Denmark).

### Ethics Statement

All animal work was conducted according to institutional guidelines and approved by the Danish Animal Experiment Inspectorate license number 2002/561-495. Mice were housed in an environmentally controlled sterile facility, exposed to a 12 hour light/dark cycle and provided with autoclaved food and water *ad libitum*.

### Statistical Analyses

A two-tailed t-test was applied to analyze gene expression data. A p-value of <0.05 was used as a threshold for statistical significance. Chalkley count data concerning microvascular density was statistically compared using Mann-Whitney and Kruskall-Wallis tests. The statistical software R r2.12.1 (http://www.R-project.org) was used to determine Ripley's K function for endothelial cell distribution on decellularized matrix.

## Supporting Information

Movie S1 Time-lapse phase contrast photomicrography of the cord-morphogenesis induced within 72 hours by serum deprivation (at 0 hours) in a monolayer of hMSC-TERT20-BD11 cells.
(MP4)
